# Immune dysfunction mediated by the competitive endogenous RNA network in fetal side placental tissue of polycystic ovary syndrome

**DOI:** 10.1371/journal.pone.0300461

**Published:** 2024-03-21

**Authors:** Ningning Xie, Fangfang Wang, Danqing Chen, Jue Zhou, Jian Xu, Fan Qu

**Affiliations:** 1 Department of Obstetrics and Gynecology, International Institutes of Medicine, The Fourth Affiliated Hospital, Zhejiang University School of Medicine, Yiwu, Zhejiang, China; 2 Women’s Hospital, School of Medicine, Zhejiang University, Hangzhou, Zhejiang, China; 3 College of Food Science and Biotechnology, Zhejiang Gongshang University, Hangzhou, Zhejiang, China; Université Clermont Auvergne - Faculté de Biologie, FRANCE

## Abstract

Polycystic ovary syndrome (PCOS), a common endocrine and metabolic disorder affecting women in their reproductive years. Emerging evidence suggests that the maternal-fetal immune system is crucial for proper pregnancy. However, whether immune function is altered at the end of pregnancy in PCOS women and the underlying molecular mechanisms is currently unexplored. Herein, the basic maternal immune system was investigated (n = 136 in the control group; n = 103 in the PCOS group), and whole-transcriptome sequencing was carried out to quantify the mRNAs, miRNAs, and lncRNAs expression levels in fetal side placental tissue of women with PCOS. GO, KEGG, and GSEA analysis were employed for functional enrichment analysis. The process of identifying hub genes was conducted utilizing the protein-protein interaction network. CIBERSORT and Connectivity Map were deployed to determine immune cell infiltration and predict potential drugs, respectively. A network of mRNA-miRNA-lncRNA was constructed and then validated by qRT-PCR. First, red blood cell count, neutrophil count, lymphocyte count, hypersensitive C-reactive protein, and procalcitonin were significantly elevated, while placental growth factor was hindered in PCOS women. We identified 308 DEmRNAs, 77 DEmiRNAs, and 332 DElncRNAs in PCOS samples. Functional enrichment analysis revealed that there were significant changes observed in terms of the immune system, especially the chemokine pathway. Eight genes, including *FOS*, *JUN*, *EGR1*, *CXCL10*, *CXCR1*, *CXCR2*, *CXCL11*, and *CXCL8*, were considered as hub genes. Furthermore, the degree of infiltration of neutrophils was dramatically decreased in PCOS tissues. In total, 57 ceRNA events were finally obtained, and immune-related ceRNA networks were validated. Some potential drug candidates, such as enalapril and RS-100329, could have a function in PCOS therapy. This study represents the inaugural attempt to evaluate the immune system at the end of pregnancy and placental ceRNA networks in PCOS, indicating alterations in the chemokine pathway, which may impact fetal and placental growth, and provides new therapy targets.

## Introduction

Polycystic ovary syndrome (PCOS) is a prevalent illness that impacts women of reproductive age. It is distinguished by the existence of hyperandrogenism, menstrual irregularities, and the formation of polycystic ovaries [1]. Based on a recent meta-analysis involving 69 studies, approximately 10% of Chinese women of reproductive age are suffering from PCOS [2]. Moreover, another meta-analysis showed that PCOS is linked to a significantly elevated prevalence of obstetric and neonatal complications when contrasted with non-PCOS controls [3]. It is essential to highlight that PCOS women may exhibit a heightened susceptibility, ranging from two to four times to experiencing preeclampsia, pregnancy-induced hypertension, spontaneous preterm labor, gestational diabetes, and an elevated likelihood of requiring cesarean delivery [4–6]. Besides being the primary factor contributing to the occurrence of absence or irregularity in menstrual cycles and infertility due to lack of ovulation, PCOS is correlated with an elevated likelihood of cardiovascular disease, type 2 diabetes mellitus, obesity, and specific types of cancer [7, 8]. The diagnostic criteria for PCOS primarily consist of the guidelines put forth by the National Institutes of Health (NIH) [9], the 2003 Rotterdam Consensus raised by the European Society of Human Reproduction and Embryology (ESHRE), and the American Society for Reproductive Medicine (ASRM) [7] and the criteria proposed by Androgen Excess Society (AES) [10]. Among them, the Rotterdam definition represents the prevailing and extensively adopted classification system for PCOS [11].

The maternal immune system function is essential in facilitating the progression of a viable pregnancy. Various immune cells and molecules are critical in placental and fetal maintenance and function [12]. A good balance between anti-inflammatory and pro-inflammatory effects is needed. The placenta, an essential component for fetal development, is the initial organ to develop in mammals. The maternal-placental-fetal units can adequately meet the requirements of the developing conceptus while providing simultaneous support to the mother [13, 14]. Numerous studies have shown that abnormal function of the placenta is linked to a variety of major obstetric syndromes, including, but not limited to, miscarriage, preeclampsia, FGR, and preterm labor [15–18]. At present, our understanding of the pathologic state of the placenta in PCOS is limited. This is attributed to the heterogeneity of the PCOS phenotype, as well as other comorbidities, including obesity, diabetes, and hypertension. A limited number of studies demonstrated that The PCOS placenta exhibited a notable decrease in placental weight, density, thickness, and volume [19]. Additionally, it was noted that women diagnosed with PCOS exhibited elevated occurrences of funisitis, chorioamnionitis, vascular thrombosis, villitis, infarction, and villous immaturity when compared to individuals without PCOS [20]. An increasing body of scholarly literature has indicated that alterations in the placenta-associated gene expression may result in abnormalities in their function and pregnancy complications [21–24]. However, the immune characteristics of peripheral blood and placental immune function throughout pregnancy in PCOS women have not been investigated in depth yet.

Over the last decade, significant advancements in high-throughput sequencing technology and various RNAs that play crucial roles in disease have been revealed [25]. In the mammalian genome, merely a minute proportion is transcribed into mRNAs that encode proteins, and the great majority produces non-coding RNAs (lncRNAs), including miRNA, lncRNA, and circular RNA (circRNA) [26]. An increasing amount of scholarly research emerged that non-coding RNAs (ncRNAs) were the key regulator of many female diseases, such as endometrial cancer, ovarian cancer, endometriosis, preeclampsia, and also, without a doubt, PCOS [27–30]. A growing amount of research suggests that ncRNAs result in the pathogenesis and progression of PCOS. Numerous markedly distinct ncRNA expressions were detected in granulosa cells (GCs), serum, follicular fluid (FF), and FF exosomes when contrasting PCOS women to those without PCOS [31–33]. A paper was conducted to identify the lncRNAs’ and mRNAs’ profiles present in exosomes. The study also aimed to uncover the pathways linked to PCOS, encompassing the MAPK and inflammation-related pathways [34]. Thus, the differential expression of ncRNAs in PCOS women may be an effective marker for PCOS diagnosis [35].

The academic community has recently shown considerable interest in the phenomenon of competitive endogenous RNA (ceRNA). It is hypothesized that mRNAs and ncRNAs cross-talk with and regulate each other through the bridge miRNA based on microRNA response elements (MREs) [36, 37]. Many researchers have noticed that ceRNA interactions play important roles in several complicated diseases, encompassing immune-related diseases [38] and cardiovascular diseases [39]. Significantly, ceRNA also assumes a pivotal role in the domain of women’s reproductive health [40], including endometriosis [41], fetal and organ development [42], spontaneous abortion [43], intrahepatic cholestasis and eclampsia in pregnancy [44, 45]. Previous studies have been conducted to construct competing ceRNA networks in PCOS. A lncRNA-miRNA-mRNA network was developed utilizing the granulosa cell profile in PCOS, which unveiled significant pathways associated with oxidative stress, inflammation, and apoptosis [46]. A separate investigation was conducted with the objective of establishing ceRNA networks through the utilization of exosomal lnRNA and circRNA. This study also elucidated the potential functions of exosomes of follicular fluid (FF) in relation to PCOS [47]. Although there were some ceRNA network studies in some tissues or cells of PCOS, unfortunately, up to now, to our knowledge, the ceRNA networks in placenta tissue of PCOS have not been explored. Therefore, a comprehensive, systematic, and in-depth exploration of the changes that occur in the placental tissue in PCOS can help to reveal the molecular mechanisms underlying pregnancy and adverse outcomes in PCOS.

In the present investigation, the alteration of the immune system of PCOS women at the end of pregnancy was investigated, and the differentially expressed lncRNAs (DElncRNAs), DEmiRNAs, and DEmRNAs were found deploying whole transcriptome sequencing of fetal side placental tissues of PCOS. The DElncRNA-DEmiRNA-DEmRNA ceRNA networks were created depending on the principles of ceRNA. Some DERNAs involved in the ceRNA network were chosen to verify by quantitative reverse transcription polymerase chain reaction (qRT-PCR). These findings helped unravel the underlying mechanisms of the ceRNA network in the physiological and pathological processes in PCOS and in finding novel targets for the development of treatments.

## Materials and methods

### Patients and tissue preparation

Between June 1st, 2019 and June 1st, 2020, typically, 103 PCOS women and 136 healthy control women who had only one female fetus at the time of cesarean delivery were recruited at the Women’s Hospital, School of Medicine, Zhejiang University. The individuals included in this study were identified as having PCOS according to the diagnostic criteria outlined in the Rotterdam Consensus, a set of guidelines jointly established by the European Society of Human Reproduction and Embryology and the American Society for Reproductive Medicine [7]. The female participants within the control group demonstrated consistent menstrual cycles while retaining typical levels of sex hormones prior to conception. All women had normal uterine and ovarian structures. The exclusion criteria encompassed various diseases that exhibit comparable clinical manifestations, encompassing androgen-secreting tumors originating from the ovary or adrenal gland, congenital adrenal hyperplasia, thyroid dysfunction, Cushing’s syndrome, primary ovarian insufficiency, hyperprolactinemia, functional hypothalamic amenorrhea, and premature ovarian failure. Peripheral blood, cord blood, and placenta samples were obtained throughout the cesarean delivery procedure. The current investigation received approval from the Ethics Committee of the Women’s Hospital, School of Medicine, Zhejiang University. The consent was informed, and the individuals in this study have given written informed consent to use the samples and data details.

Following obtaining informed consent from the patients, we collected placental tissue from the term fetal side of those women who underwent cesarean section deliveries, as described in a previous study. Immediately after the placental tissue was stripped, it was snap-frozen in liquid nitrogen for 30 min, after which it was stored at –80°C.

### Laboratory investigations

Complete blood counts encompassing red blood cells, white blood cells, lymphocytes, neutrophils, eosinophils, monocytes, and basophils were performed using whole blood. The biochemical tests were performed using the serum samples, including procalcitonin, hypersensitive C-reactive protein (hs-CRP), and placental growth factor (PLGF). The test results were obtained from hospital records.

### RNA extraction and quality control

Total RNA extraction was carried out by deploying TRIzol reagent (Invitrogen) from fetal side placental tissue. Typically, five specimens were encompassed in each group, and the extraction protocol provided by the manufacturer was followed. A Bioanalyzer 2100 (RRID: SCR_019715) and RNA 6000 Nano LabChip Kit (Agilent, Santa Clara, CA, USA) were employed to assess the amount and purity of total RNA. RNA with RNA integrity number (RIN) ≥ 7 and 28S/18S ≥ 0.7 was considered to be high-quality RNA.

### Whole transcriptome sequencing

Two libraries were constructed for the purpose of whole transcriptome sequencing. The TruSeq Small RNA Sample Prep Kits (Illumina, San Diego, USA) were employed to create the small RNA library. A 1 μg of total RNA was utilized in accordance with the provided protocol. Following the completion of library preparation, the created libraries underwent sequencing by employing the Illumina Hiseq2000 platform, employing a single-end configuration with a read length of 1×50 bp. For the mRNA and circRNA libraries, first, ribosomal RNA was depleted, and the remaining RNA was fragmented. Following the purification process, the poly(A)–or poly(A)+ RNA fractions were subjected to fragmentation by means of divalent cations at an increased temperature. Additionally, the cDNA library was constructed utilizing the mRNA-Seq sample preparation kit (Illumina, San Diego, CA, USA). Finally, the paired-end sequencing was conducted on the Illumina NovaSeq 6000 system with a 2 × 150 bp read length (LC Bio, China).

### Identification of DElncRNAs, DEmiRNAs, and DEmRNAs

To perform analysis on mRNAs, the initial step involved utilizing Cutadapt (RRID: SCR_011841) and Perl scripts to eliminate reads containing adaptor contamination, low-quality bases, and unclear bases [48]. Then, FastQC (http://www.bioinformatics.babraham.ac.uk/projects/fastqc/, RRID: SCR_014583) was employed for the purpose of confirming the sequence quality. The standard-compliant reads were mapped to the human genome by Bowtie2 (RRID: SCR_016368) and Tophat2 [49, 50]. The assembled mapped reads of each specimen were processed by deploying StringTie (RRID: SCR_016323) [51]. The determination of transcript expression values was achieved through the computation of fragments per kilobase of exon per million reads mapped (FPKM) by deploying the software tools StringTie and Ballgown [52]. Finally, the DEmRNAs were screened out with |log2 (fold-change)| ≥ 1 and P-value < 0.05 by edgeR [53].

For lncRNAs analysis, following the reads assembly using StringTie, transcripts smaller than 200 bp, as well as known mRNAs, were subsequently excluded from subsequent analysis. Subsequently, the remaining transcripts were subjected to lncRNA prediction. The utilized prediction software consisted of the Coding Potential Calculator (CPC), the Coding-Non-Coding Index (CNCI), and the Coding Potential Assessment Tool (CPAT) [54–56]. Transcripts that demonstrate the ability to encode protein were categorized as newly discovered mRNA and subsequently designated as such through the implementation of filtration methodologies. The FRKM method was employed to determine the lncRNA expression level. Subsequently, DElncRNAs were identified using a set of criteria that included a two-fold change and a P-value below 0.05.

For miRNA analysis, the ACGT101-miR software, which was self-developed by LC Sciences, was used. The analytical procedure of this software can be outlined in the following manner: (1) The clean data were acquired through the elimination of 3’ junction and extraneous sequences; (2) Sequences ranging from 18–26 nucleotides in length were preserved; (3) Non-miRNA sequences, such as rRNA, tRNA, snRNA, and snoRNA, were eliminated by cross-referencing with mRNA, RFam, and Repbase databases; (4) Valid data was retrieved and subsequently contrasted to precursors and genomes to identify miRNAs; (5) If |log2FC| ≥1 and P-value < 0.05, the miRNAs were deemed as differently expressed miRNAs (DEmiRNAs).

The DEmRNAs, DElncRNAs, and DEmiRNAs were visualized by volcano plots and heatmaps [57].

### Functional enrichment analysis of DEmRNAs

To further ascertain the DEmRNAs bio-function, the Kyoto Encyclopedia of Genes and Genomes (KEGG) pathway and GO enrichment analysis, encompassing biological process (BP), cellular component (CC), and molecular function (MF), were executed using R package clusterProfiler v3.10.1 [58, 59]. Gene Set Enrichment Analysis (GSEA) analysis was conducted on the normalized gene expression dataset with the KEGG dataset from c2.cp.kegg.v7.4 using clusterProfiler V3.10.1. A P-value < 0.05 was deemed to be significant [60].

### Hub genes identification and module analysis

The Protein-protein interaction (PPI) network was created with a confidence score ≥ 0.7 by the STRING online database (https://cn.string-db.org/; version 11.5) to determine the hub genes in these DEmRNAs. The Cytoscape plug-in app Cytohubba was used to find important nodes in biological networks [61]. The outcomes visualization used Cytoscape software (version 3.9.1) [62]. In order to examine the clustering modules of greatest importance, we employed the Metascape platform (http://metascape.org; V3.5.20230101), an online tool designed for comprehensive analyses [63].

### Assessment of immune cell infiltration

CIBERSORT is a computational technique utilized for the assessment of immune cell presence and abundance within intricate biological tissues depending on the analysis of gene expression patterns [64]. The abundance of immune infiltrating cells was quantified depending on the mRNA profile in the control and PCOS group. P value was determined employing the Mann-Whitney U test. A percentage bar graph was deployed for the purpose of showing the immune cell composition in each group. Boxplots were utilized to show the disparities in these cells between the control and PCOS specimens.

### Correlation analysis between hub DEmRNAs and infiltrating immune cells

Pearson correlation analysis was conducted by deploying R package stats V4.0.3 to evaluate the interactive connection between 22 immune cells and the association between hub genes and the content of immune cells.

### Sankey diagram

The Sankey plot was used to depict the selected ceRNA network (lncRNA-miRNA-mRNA). The R package ggalluvial V3.6.1 was utilized to generate the Sankey plot [65].

### LncRNA-mRNA co-expression network analysis

LncRNAs often participate in the regulation of nearby mRNAs through cis-regulation. The cis-regulation of lncRNA was forecasted through the examination of the co-expression network between lncRNA and mRNA. Hence, the Pearson correlation coefficient was deployed for the purpose of ascertaining the association between the lncRNA and mRNA expression levels. P-value < 0.05 was considered significant. LncRNA cis-regulatory target genes are predicted mainly based on positional relationships, defining the presence of DElncRNAs with DEmRNAs within 100 kb upstream and downstream in the chromosome.

### Construction of lncRNA–miRNA–mRNA ceRNA network

First, two individual pairs, lncRNA–miRNA and miRNA–mRNA pairs, were constructed using two algorithms (TargetScan5.0 and miRanda3.3a) [66, 67]. The target pairs were selected according to the criteria that the TargetScan score is ≥ 90 and the miRanda Energy is < -20. Subsequently, to create a ceRNA network, we selected DElncRNAs and DEmRNAs that exhibit binding affinity towards the same DEmiRNA. This ceRNA network was constructed employing Cytoscape 3.9.1.

### Identification of potential small-molecule drug

For the purpose of finding the possible drugs for the PCOS treatment, we used a web tool called Clue (the updated Connectivity Map; https://clue.io.) [68]. The web tool was employed to upload the list of up-regulated DEmRNAs. L1000, a highly reproducible method, was selected to determine the connections between drugs and genes. Ultimately, the top 15 drugs were considered as candidate drugs.

### Quantitative real-time PCR verification

The RNA expressions involved in the ceRNA network in each group were verified by qRT-PCR. For mRNA and lncRNA verifications, PrimeScript™ RT reagent Kit with gDNA Eraser (Perfect Real Time) kit (TaKaRa, RR047A, Japan) was deployed in reverse transcription procedure. Subsequently, qPCR was conducted, and TB Green® Premix Ex Taq ™ (Tli RNaseH Plus) (TaKaRa, RR420A, Japan) was deployed. The miRNA verification process involved the utilization of the miDETECT A TrackTM miRNA qRT-PCR Starter Kit (C10712, RIBOBIO, China) and the miDETECT A TrackTM miRNA qPCR Kit (C10711, RIBOBIO, China). The quantification of mRNA, lncRNA, and miRNA expression levels was conducted utilizing the 2- ^ΔΔCT^ approach. ACTB and GAPDH were used as reference genes for mRNA and lncRNA, U6 and 5S were used as reference genes for miRNA.

### Statistical analysis

The bioinformatic analysis was conducted utilizing the OmicStudio tools available at https://www.omicstudio.cn/tool [69]. The qRT-PCR data underwent statistical analysis using GraphPad Prism software (version 9.2.0). A significance level of P < 0.05 was deemed to denote significance.

## Results

### The anthropometric and laboratory investigations

Table 1 lists the anthropometric and laboratory profiles of both control and PCOS groups. The age and BMI were similar between the two groups. Regarding the hematological parameters, red blood cell count, neutrophil count, and lymphocyte count were significantly elevated in PCOS women compared to the controls. Moreover, PCOS women exhibited markedly heightened hs-CRP and procalcitonin concentrations but lower levels of PLGF. There was an increased likelihood of neonatology transfer among the progeny of women with PCOS (46.60% vs. 24.26%).

**Table 1:** The anthropometric and laboratory investigations of the polycystic ovary syndrome (PCOS) and control groups.

Parameters	Controls (n = 136)	PCOS (n = 103)	*p* value
Age (years)	32.40 ± 3.45	31.76 ± 4.09	0.192
Menstrual cycle (days)	29.40 ± 2.03	52.37 ± 15.79	**0.000** ^*^
Total testosterone (nmol/L)	0.70 ± 0.33	1.46 ± 0.56	**0.000** ^*^
Progestational BMI (kg/m2)	21.18 ± 2.67	21.73 ± 3.23	0.156
BMI at delivery (kg/m^2^)	26.98 ± 2.92	27.04 ± 3.38	0.895
WBC count (10^9^/L)	8.16 ± 1.85	8.53 ± 2.61	0.202
RBC count (10^12^/L)	3.94 ± 0.37	4.28 ± 1.52	**0.013** ^*^
Neutrophils (%)	73.72 ± 5.52	73.76 ± 6.47	0.956
Neutrophils count (10^9^/L)	6.07 ± 1.70	6.63 ± 2.27	**0.030** ^*^
Lymphocytes (%)	17.71 ± 4.66	17.85 ± 5.44	0.835
Lymphocytes count (10^9^/L)	1.40 ± 0.35	1.50 ± 0.42	**0.035** ^*^
Monocytes (%)	7.55 ± 1.77	7.24 ± 1.87	0.189
Monocytes count (10^9^/L)	0.61 ± 0.18	0.63 ± 0.22	0.397
Eosinophils (%)	0.74 ± 0.56	0.80 ± 0.66	0.439
Eosinophils count (10^9^/L)	0.11 ± 0.60	0.07 ± 0.07	0.537
Basophils (%)	0.29 ± 0.20	0.28 ± 0.14	0.716
Basophils count (10^9^/L)	0.02 ± 0.02	0.02 ± 0.02	0.643
Hs-CRP (mg/L)	3.58 ± 2.80	5.18 ± 4.47	**0.001** ^*^
Procalcitonin (ng/mL)	0.08 ± 0.05	0.12 ± 0.11	**0.032** ^*^
PLGF (pg/mL)	548.63 ± 509.77	346.46 ± 309.33	**0.044** ^*^
Neonatal weight (g)	3321.62 ± 469.63	3246.96 ± 592.73	0.280
Neonatal transfer rate (%)	24.26	46.60	—

BMI: body mass index; WBC: white blood cell; RBC: red blood cell; hs-CRP: hypersensitive C-reactive protein; PLGF: placental growth factor. Data are presented as mean ± standard deviation (SD). A p-value of < 0.05 was considered statistically significant.

### Identification of DEmRNAs, DElncRNAs, and DEmiRNAs

**Fig 1:**
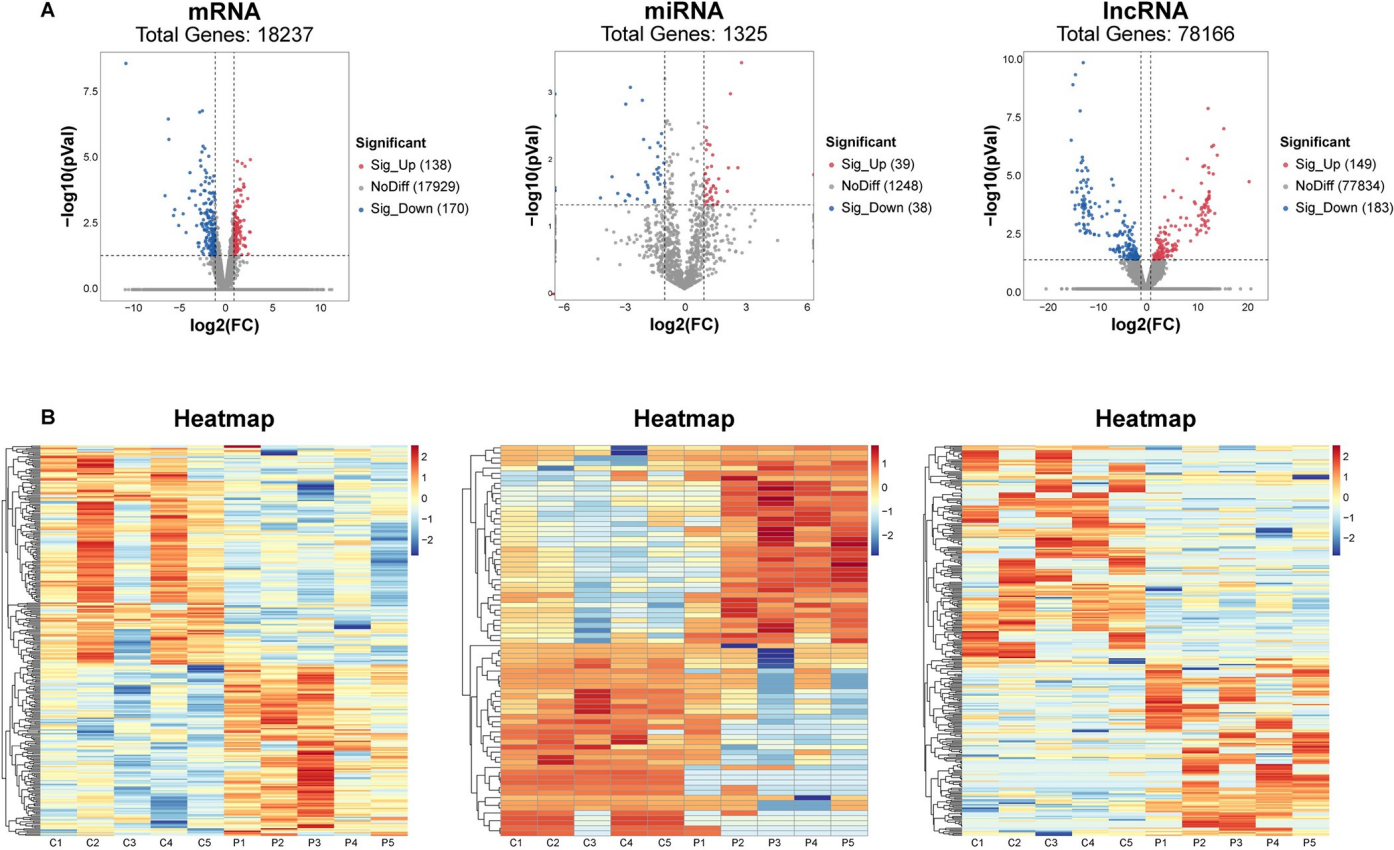
Identification of differentially expressed RNAs (DERNAs) in fetal side placental tissue of PCOS and control group. (A) Volcano plots of DERNAs with log_2_ (foldchange) as the abscissa and -log10 (P value) as the ordinate. The RNAs that exhibited significant up- or down-regulation in PCOS are symbolically represented by red and blue splashes, respectively (|log_2_FC| ≥ 1, P < 0.05). Gray splashes represent RNAs whose expression is not significantly altered. (B) Heatmaps for DEmRNAs, DEmiRNAs, and DElncRNAs in PCOS and control group (n = 5 in each group). Each row in the dataset corresponds to a specific RNA molecule, while each column represents a unique sample. The color red is indicative of a heightened level of expression, while the color blue signifies a diminished level of expression.

Ten tissues (including five normal and five PCOS placentas) were deployed for the whole transcriptome analysis. Based on the established screening criteria, a comprehensive set of 308 DEmRNAs (138 up-regulated and 170 hindered), 332 significant DElncRNAs (149 elevated and 183 hindered), and 77 significant DEmiRNAs (39 elevated and 38 hindered) were acquired and displayed by volcano plot (Fig 1A) and heatmaps (Fig 1B), respectively.

### Functional enrichment analysis of DEmRNAs

**Fig 2:**
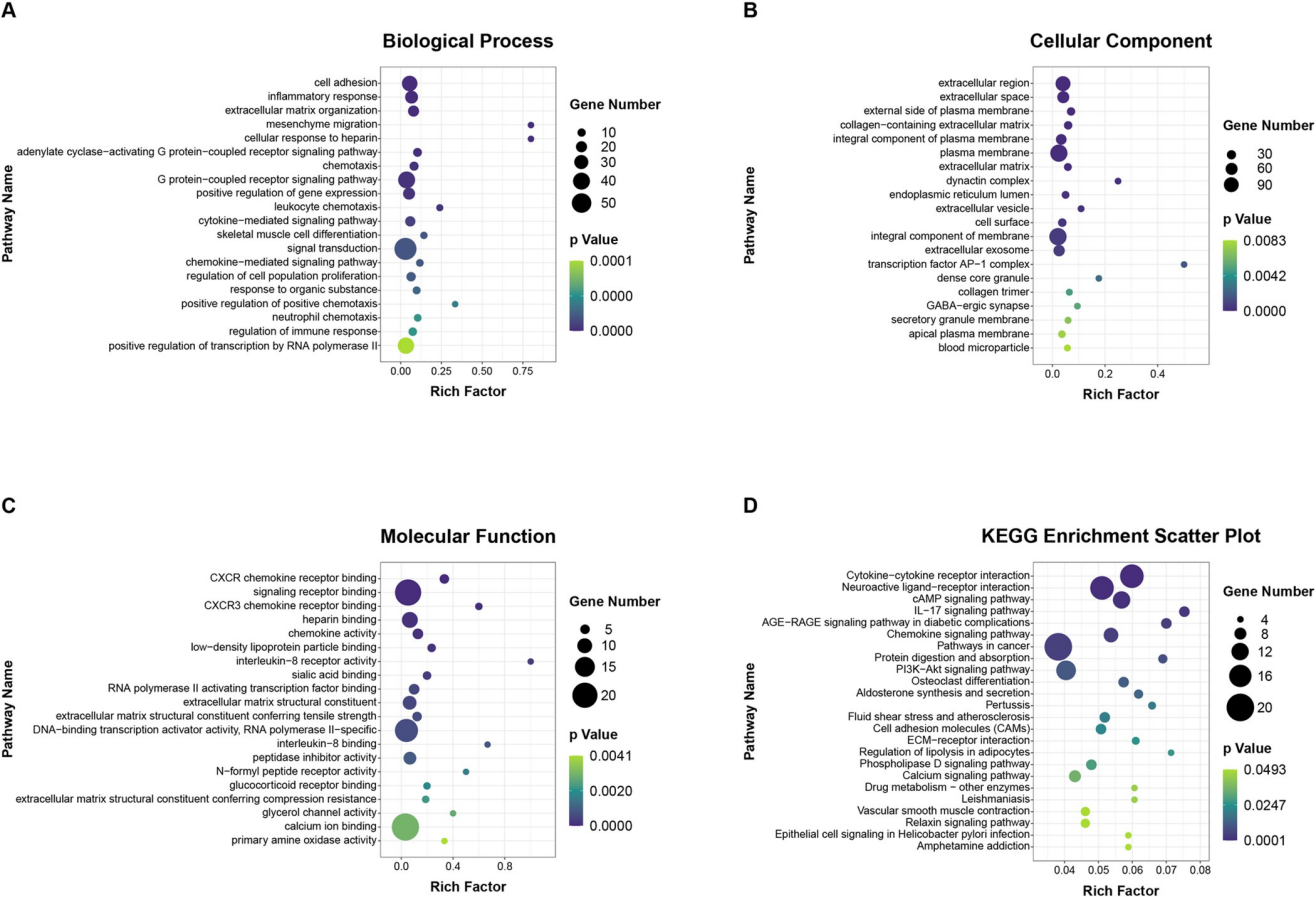
Functional enrichment analysis of DEmRNAs in fetal side placental tissue of PCOS and control group. The scatter plot of enriched top 20 Gene Ontology (GO) terms in (A) biological process (BP), (B) cell components (CC), and (C) molecular function (MF). (D) Kyoto Encyclopedia of Genes and Genomes (KEGG) pathway enrichment scatter plot of DEmRNAs in PCOS and control group. The x-axis denotes the rich factor, while the y-axis corresponds to the pathway nomenclature. The dot size corresponds to the gene number, and dot color indicates the P-value. All the significantly enriched KEGG pathways are shown.

Moreover, to examine the possible functional functions of these DEmRNAs in PCOS, GO, KEGG, and GSEA analysis was conducted. Fig 2 displays the top 20 BP, CC, and MF-GO terms. Specifically, for BP terms, DEmRNAs exhibited substantial improvements primarily in terms related to the immune system, encompassing inflammatory response, chemotaxis, leukocyte chemotaxis, cytokine-mediated pathway, chemokine-mediated pathway, positive regulation of positive chemotaxis, neutrophil chemotaxis, and regulation of immune response. For CC terms, the terms of extracellular region and extracellular space were most significantly changed. Moreover, the transcription factor AP-1 complex term was most enriched (S1A Fig). Notably, immune-related terms were also involved in MF terms, like CXCR chemokine receptor binding, CXCR3 chemokine receptor binding, chemokine activity, interleukin-8 receptor activity, and interleukin-8 binding.

KEGG pathway analysis emerged that these DEmRNAs to be improved in immune system (IL-17 signaling pathway, chemokine pathway), endocrine system (aldosterone synthesis and secretion, regulation of lipolysis in adipocytes, relaxin pathway); molecules and interaction (cytokine-cytokine receptor interaction, neuroactive ligand-receptor interaction, cell adhesion molecules (CAMs), ECM-receptor interaction); signal transduction (cAMP pathway, PI3K-Akt pathway, phospholipase D pathway, calcium pathway); and infectious disease (pertussis, leishmaniasis, epithelial cell signaling in Helicobacter pylori infection). Fig 2D shows all significantly enriched pathways.

In addition, GSEA was performed in the DEmRNAs between control and PCOS groups. Interestingly, immune-related pathways showed remarkable enrichment, including natural killer cell-mediated cytotoxicity, autoimmune thyroid disease, systemic lupus erythematosus, B cell receptor pathway, Fc gamma R-mediated phagocytosis, complement and coagulation cascades, and intestinal immune network for IgA generation (S1B and S1C Fig) (S1 Table).

### PPI network construction and hub gene identification

**Fig 3:**
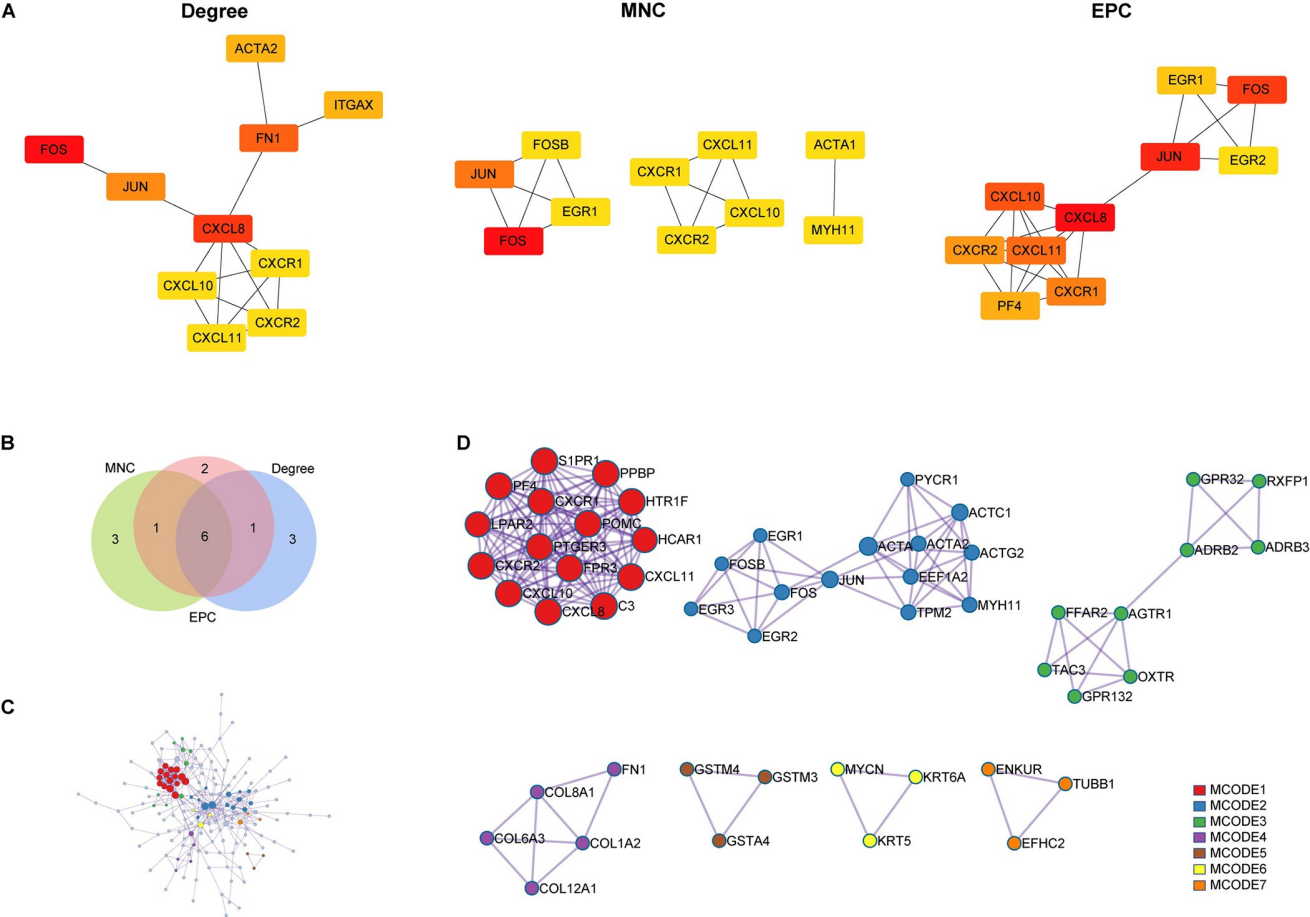
Protein-protein interaction (PPI) network creation and hub gene identification. (A) Top 10 hub genes obtained by three different algorithms (Degree, MNC, and EPC) using Cytohubba plug-in app. MNC: Maximum Neighborhood Component; EPC: Edge Percolated Component. Darker colors indicate higher rankings. (B) Venn diagram of the hub genes from Degree, MNC, and EPC algorithms. (C) PPI network constructed by Metascape analysis of DEmRNAs. (D) Seven sub-network modules were aggregated and extracted from PPI network.

To fully understand the key genes in PCOS pathogenesis, the PPI network of DEmRNAs was created by the STRING website with a confidence score of 0.70. Three hundred and eight DEmRNAs were submitted to the STRING database, and the network was composed of 119 nodes and 162 edges. CytoHubba, a plug-in that uses multiple topological algorithms in Cytoscape, was employed to screen out hub genes. The intersection of the top 10 genes derived from three distinct algorithms, namely MNC, EPC, and degree, is computed. Additionally, those genes that were shared by at least two algorithms were considered hub genes. Fig 3A displays the top 10 genes found by three algorithms. The shared genes were shown in a Venn diagram (Fig 3B). Finally, eight genes, including *FOS*, *JUN*, *EGR1*, *CXCL10*, *CXCR1*, *CXCR2*, *CXCL11*, and *CXCL8*, were considered as hub genes.

### Module extraction in Metascape

A clustering analysis was utilized to reveal the core functions in DEmRNAs. In the results of Metascape analysis, seven sub-network modules were aggregated and extracted (Fig 3C and 3D). 3Among these modules, the top three show a complicated network. MCODE1 contained 15 nodes and 104 edges, in which the immune-related mRNAs were most included, such as *CXCL10*, *CXCR1*, *CXCR2*, *CXCL11* and *CXCL8*. MCODE2 contained 14 nodes and 41 edges, in which some transcription factors were involved, including *JUN*, *FOS*, *FOSB*, *EGR1*, *EGR2*, and *EGR3*. MCODE3 contained 9 nodes and 16 edges, enriched by hormone and lipid metabolism-related genes.

The above results demonstrated that the immune-related pathways and hub genes exhibited significant dissimilarities between the PCOS and control groups.

### Immune cell infiltration analysis

**Fig 4:**
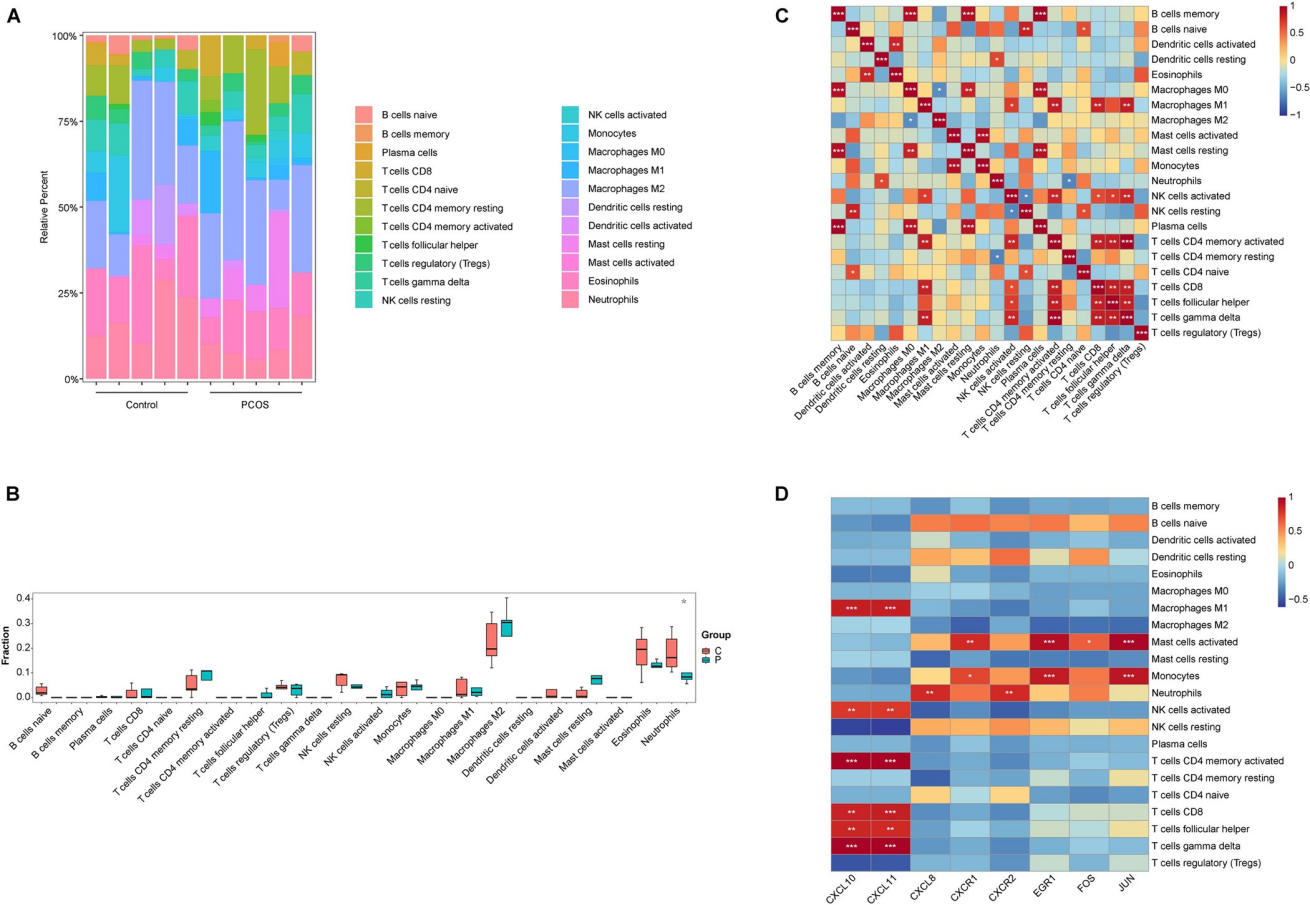
Landscape of immune cell infiltration in fetal side placental tissue of PCOS and control group. (A) Bar plot of the proportion of 22 infiltrated immune cells between PCOS and control group. Each color represents a type of immune cell. (B) The violin plot depicted the differences in immune infiltrating between PCOS and normal samples (n = 5 in each group). (C) Heatmap of correlations among 22 types of immune cells. (D) Correlation analysis between eight hub genes and 22 infiltrated immune cells. The colors blue and red are employed to symbolize positive and negative correlation, respectively. The darker the color, the greater the connection. *P < 0.05, **P < 0.01, ***P < 0.001.

To further explain the difference in immune systems between PCOS women and healthy controls, immune infiltration analysis was conducted using the CIBERSORT algorithm. First, the number of 22 types of infiltrating immune cells in placental tissues was calculated and shown in the bar plot (Fig 4A). The boxplot indicated the difference between each immune cell in control and PCOS group (Fig 4B). The outcomes emerged that the degree of Neutrophils infiltration (P = 0.021) was dramatically decreased in PCOS tissues compared with controls. Subsequently, the Pearson correlation coefficient between 22 infiltrating immune cells was calculated and illustrated in correlation heatmaps (Fig 4C). Neutrophils correlated positively with resting dendritic cells and negatively with resting memory CD4 T cells. An interesting conclusion can be drawn that the infiltrating immune cells in the fetal side placenta tissue of PCOS were remarkably changed.

### Correlation analysis between hub genes and infiltrating immune cells

Next, we analyzed the connection between hub genes and immune cells based on expression level. Fig 4D presents that neutrophils were significantly positively connected to *CXCL8* (rho = 0.828, P = 0.003), *CXCR2* (rho = 0.835, P = 0.003); *CXCL10* and *CXCL11* were significantly positively linked to 6/22 infiltrating immune cells.

### Predicted lncRNA targets and functional enrichment analysis

Moreover, to address the DElncRNAs functions, the potential cis target transcripts were identified. First, the Pearson correlation test was conducted to determine the connection of expression levels between lncRNA and mRNA. Furthermore, the FEELnc software was employed to detect the differentially expressed transcripts within a genomic region spanning 100 kb upstream and downstream of the DElncRNAs. Transcripts that meet both of these conditions were regarded as target transcripts. Ultimately, 35 lncRNA-mRNA pairs were screened. A total of 27 cis-transcripts were regulated by 30 regulatory DELncRNAs (S2 Table).

**Fig 5:**
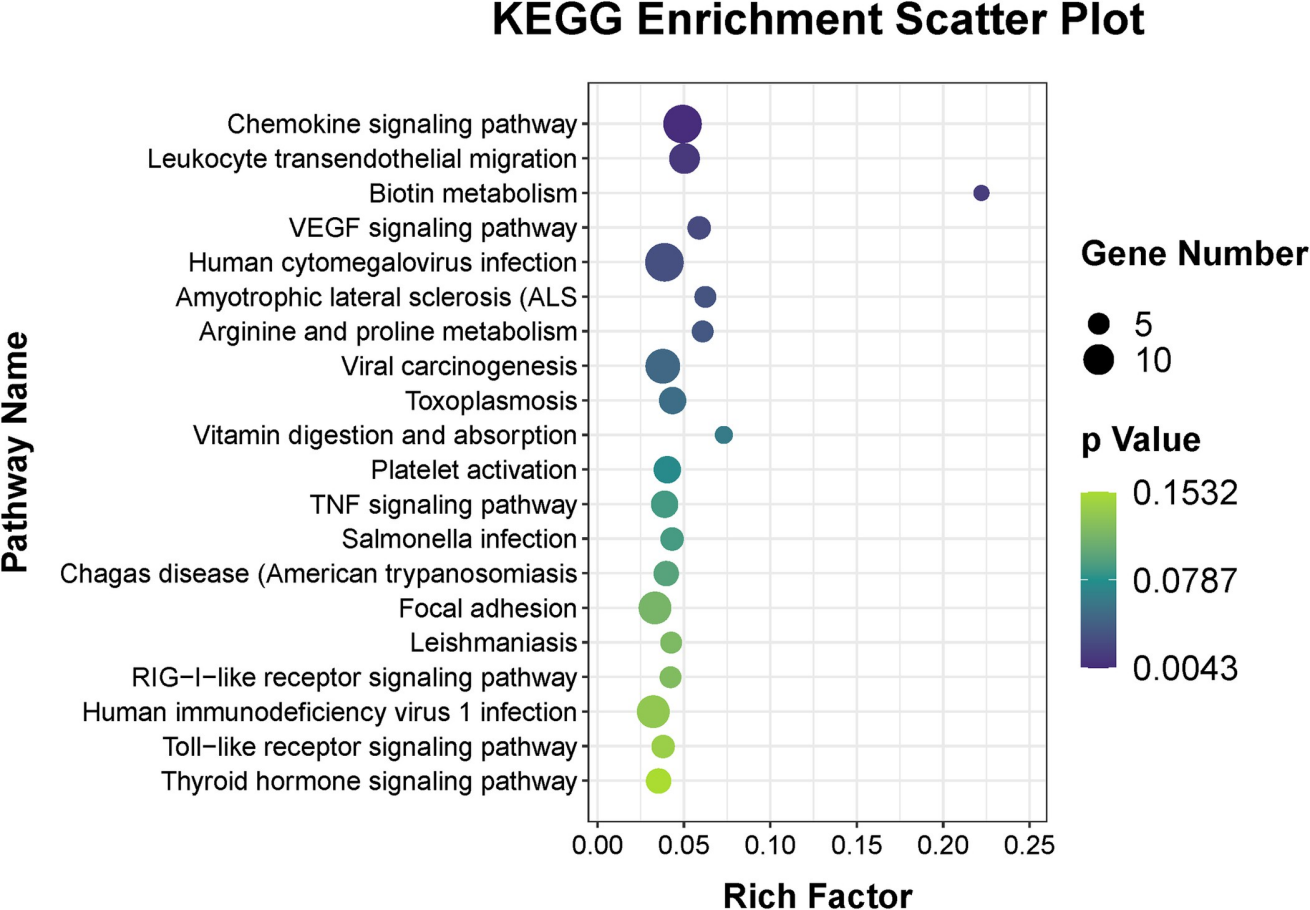
KEGG enrichment analysis of the DElncRNAs’ potential cis target transcripts. Top 20 significantly enriched KEGG pathways of DEmRNAs in PCOS and control group. The x-axis denotes the rich factor, while the y-axis corresponds to the pathway nomenclature. The dot size corresponds to the gene number, and dot color indicates the P-value.

Moreover, KEGG enrichment analysis was performed for these target transcripts. It is surprising that the most significantly changed pathways were also immune-related pathways, encompassing the chemokine signaling pathway and leukocyte transendothelial migration (Fig 5).

### Construction of the ceRNA networks

**Fig 6:**
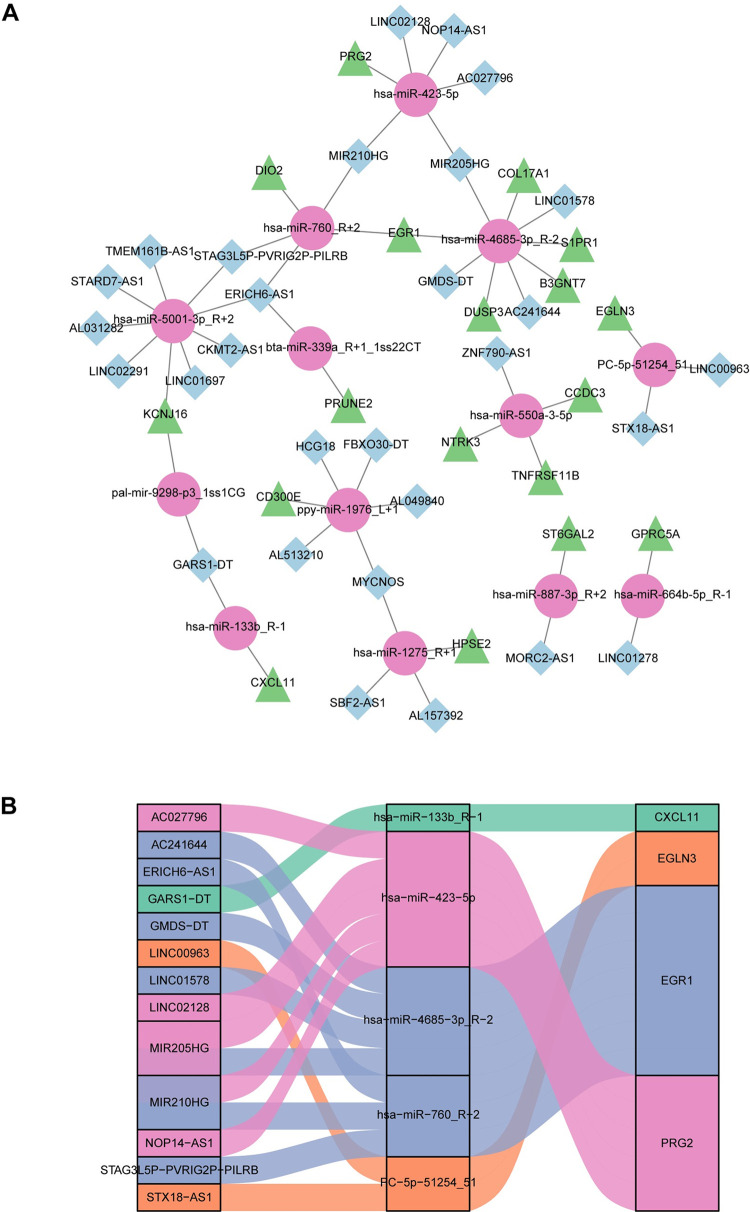
Creation of the competitive endogenous RNA (ceRNA) regulatory network. (A) The ceRNA regulatory network included 18 DEmRNAs, 13 DEmiRNAs, and 29 DElncRNAs in PCOS and control group. DEmRNAs, DEmiRNAs, and DElncRNAs are denoted as green triangles, pink circles, and blue diamonds, respectively. (B) A Sankey diagram of the ceRNA network associated with the immune system. The miRNAs interacting with them are in the middle. Each ceRNA network is represented by the same color.

After identifying the regulation relationships of DEmiRNAs-DElncRNAs and DEmiRNAs-DEmRNAs with two algorithms (TargetScan5.0 and miRanda3.3a), the DElncRNA (known lncRNA) and DEmRNA that were regulated by the same DEmiRNA were screened. As a result, 29 DElncRNAs, 13 DEmiRNAs, and 18 DEmRNAs were utilized in order to construct ceRNA networks, with the aim of investigating their respective roles in PCOS placental tissues. In total, 57 ceRNA events were finally obtained (Fig 6A). Moreover, the ceRNA networks of hub or immune mRNAs (*CXCL11*, *EGR1*, *EGLN3*, and *PRG2*) were visualized in the Sankey plot (Fig 6B). These potential ceRNA networks might provide an illuminating insight into the pathogenesis of PCOS.

### qRT‒PCR validation

**Fig 7:**
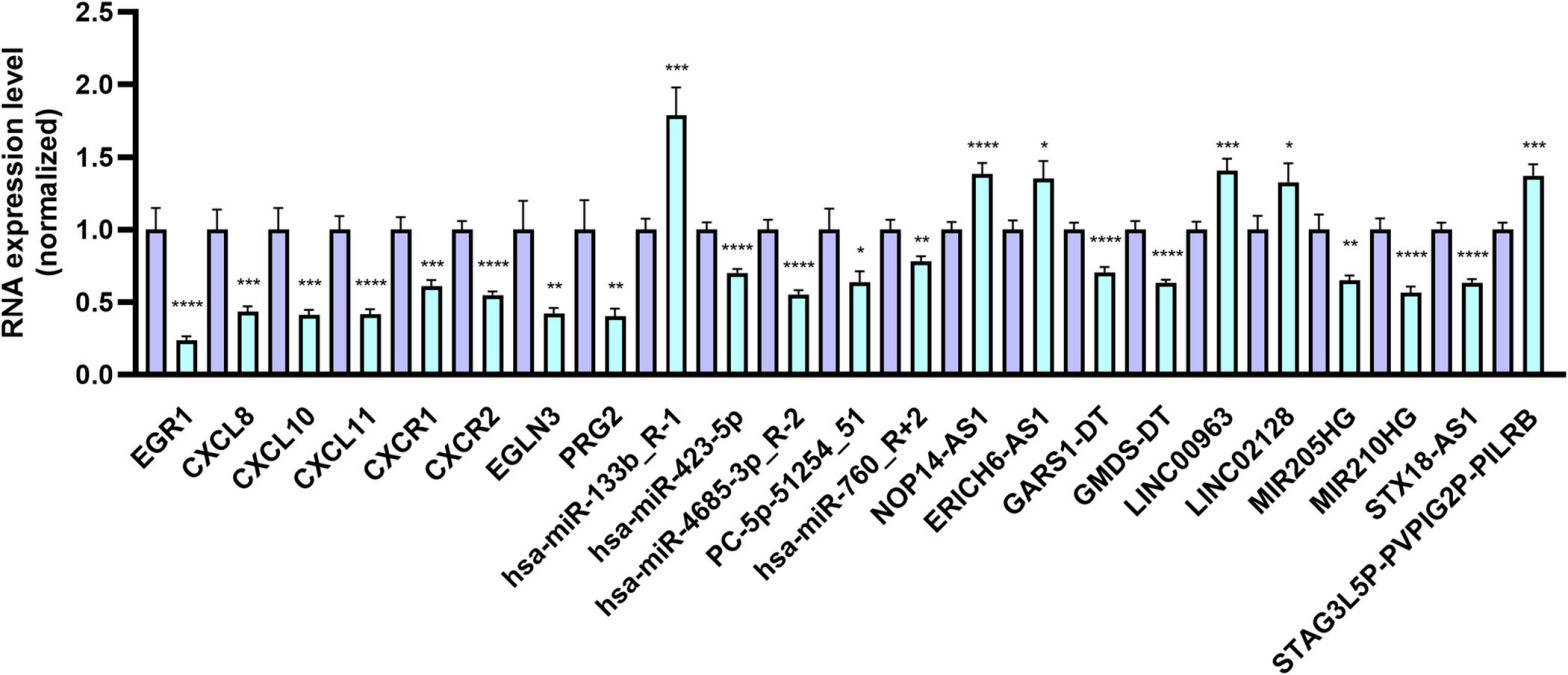
The validation of DERNAs of the ceRNA network related to the immune system in PCOS and control group using qRT-PCR. The quantification of mRNA, lncRNA, and miRNA expression levels was conducted utilizing the 2- ^ΔΔCT^ approach. ACTB was used as reference genes for mRNA and lncRNA, and U6 was used as reference genes for miRNA. Data were reported as means ± SEM; n = 30 in each group. Two-tailed student’s t-tests were used, and significant differences were considered when the P-value < 0.05. *P < 0.05, **P < 0.01, ***P < 0.001, ****P < 0.0001.

To confirm the hub mRNAs’ and DERNAs’ expression included in the ceRNA network, we measured the levels of eight mRNAs, ten lncRNAs, and five miRNAs using qRT-PCR methods. All primers are listed in S3 Table. The expression variations of the vast majority of chosen DERNAs were in accordance with the whole transcriptome sequencing data. All the immune-related mRNAs (*EGR1*, *CXCL8*, *CXCL10*, *CXCL11*, *CXCR1*, *CXCR2*, *EGLN3* and *PRG2*) were significantly decreased in PCOS group. lncRNA *NOP14-AS1*, *ERICH6-AS1*, *LINC00963*, *LINC02128* and *STAG3L5P-PVPIG2P-PILRB* were dramatically upregulated, while *GARS1-DT*, *GMDS-DT*, *MIR205HG*, *MIR210HG*, and *STX18-AS1* were down-regulated in PCOS group. Of the five selected DEmiRNAs, *hsa-miR-133b_R-1* was up-regulated, and other four DEmiRNAs (*hsa-miR-423-5p*, *hsa-miR-4685-3p_R-2*, *PC-5p-51254_51*, and *hsa-miR-760_R+2*) were downregulated in PCOS group (Fig 7 and S2 Fig).

### Prediction of potential drugs for PCOS treatment

CMap, a computational screening approach, was used to predict prospective small-molecule PCOS therapeutic drugs. A total of 138 up-regulated DEmRNAs were imported into the CMap web tool. After conducting the signature query, we identified the top 15 drugs with the greatest enrichment score. These drugs have been identified as potential therapeutic options for PCOS (S4 Table). These drugs consisted of receptor antagonists (clobetasol, aminopentamide, phentolamine, hydrocortisone-phosphate, RS-100329, brompheniramine), enzyme inhibitors (tubacin, enalapril, cilostamide, tipifarnib) and others.

## Discussion

In this paper, the alteration of the immune system of PCOS women at the end of pregnancy was investigated, and whole-transcriptome analysis of fetal side placental tissue was conducted. Many significantly changed RNAs (mRNAs, miRNAs, and lncRNAs) were found. Additionally, GO, KEGG, and GSEA analyses were conducted to find the possible functional implications of these DEmRNAs in PCOS. The outcomes from all three methods consistently demonstrated that DEmRNAs exhibited a predominant enrichment in terms linked to the immune system, particularly the inflammatory response, neutrophil chemotaxis, and CXCR chemokine receptor binding (Fig 2).

Hs-CRP and procalcitonin can be used to assess persistent inflammation. In the present paper, the hs-CRP levels and procalcitonin in the PCOS group were significantly elevated in comparison with control women at the end of pregnancy. Previous studies have also shown that circulating hs-CRP and procalcitonin are elevated in non-pregnant women with PCOS [70, 71]. Low circulating PLGF levels were linked to intrauterine growth restriction and pre-eclamptic pregnancies [72]. The PLGF levels in PCOS women at the end of pregnancy was drastically reduced, indicating abnormal placentation in the PCOS group (Table 1).

An increasing body of empirical support in the past few years has substantiated the pivotal role of chemokines and their corresponding receptors in the functionality of the immune system, angiogenesis/angiostasis, organ development, tumorigenesis, and metastasis [73]. Similarly, numerous recent studies have shown that CXC chemokines may participate in PCOS pathogenesis. Elevated levels of CXCL4 and CXCL8 have been observed in the FF of individuals with PCOS, potentially exacerbating the impairment of folliculogenesis, a prominent characteristic of PCOS [74].

To fully understand the key DEmRNAs in the pathogenesis of PCOS, hub genes were found using CytoHubba plug-in depending on the PPI network. Notably, the eight hub genes contained five chemokines or their receptors, including *CXCL10*, *CXCR1*, *CXCR2*, *CXCL11*, and *CXCL8*. Meanwhile, sub-network modules were extracted using Metascape; surprisingly, MCODE1 contained all the five hub genes (Fig 3). The chemokine *CXCL8* mediates neutrophil activity via two G protein-coupled receptors, *CXCR1* and *CXCR2* [75]. A study revealed that women with PCOS exhibited a higher CXCL8 concentration in the FF contrasted to the control [76]. The CXCL8 baseline level in the serum of women with PCOS was considerably higher, as stated by a randomized clinical trial investigation [77]. Moreover, a previous study documented a CXCL8 up-regulation in the ovarian tissue of a mouse model exhibiting PCOS [78]. The aforementioned studies have presented empirical evidence suggesting that increased levels of CXCL8 may be employed as a biomarker in PCOS treatment.

It is noteworthy that recent research has indicated an elevation in CXCL10 concentrations in FF among PCOS women. Furthermore, these elevated concentrations were found to exhibit a positive correlation with the insulin resistance states commonly associated with PCOS [79]. Moreover, a recent paper identified increased serum CXCL8 and CXCL10 levels at week 10 of pregnancy in PCOS women [80]. Nevertheless, no variations were noted in the CXCL10 levels between non-obese, non-IR PCOS women and controls [81]. The serum CXCL11 levels were strongly associated with prolactin and 17-OH-progesterone levels in PCOS [82]. Other chemokines, like CXCL1~CXCL3, also contributed to the PCOS pathogenesis [83–85]. In this paper, the *CXCL10*, *CXCR1*, *CXCR2*, *CXCL11*, and *CXCL8* expression levels were all remarkably hindered in the fetal side placental tissue in the PCOS group (Fig 7 and S2 Fig).

The placental tissue of the PCOS group exhibited a significant decrease in the three additional hub gene expression levels, namely *FOS*, *JUN*, and *EGR1* (Fig 7 and S2 Fig). FOS and JUN are constituents of the activator protein 1 (AP-1) superfamily of transcription factors, which have a significant role in various cellular processes encompassing differentiation, proliferation, hypoxia, apoptosis, angiogenesis, and steroidogenesis [86, 87]. Previous studies demonstrated that *JUN/FOS* could trans-activate *CXCL8* transcription. In particular, the *c-JUN* kinase (*JNK1*) phosphorylates *c-JUN*, leading to its translocation into the nucleus alongside *c-Fos* and promoting *CXCL8* expression [88]. A separate investigation has demonstrated that the activation of *EGR1* is imperative for the upregulation of *CXCL8* expression in BeWo cells upon exposure to palmitic acid and TNFα [89]. Single-cell analysis of trophoblast stem cell-based organoids suggested that EGR1 was a novel transcription factor that regulates cytotrophoblast biology in vivo and in trophoblast organoids [90].

With the capability of migrating towards sites of inflammation in an effort to eliminate pathogens, neutrophils comprise the majority of circulating innate immune cells in humans. Previous research demonstrated that the neutrophil count in PCOS patients was significantly greater than their corresponding controls [91]. In this study, immune cell infiltration analysis discovered that the degree of neutrophil infiltration (P = 0.021) was largely decreased in PCOS placental tissues compared with controls (Fig 4B). Maternal neutrophils have a crucial contribution in facilitating gestational tolerance and the subsequent delivery of the fetus. In a recent study, neutrophil depletion was achieved through pharmacological means by administering anti-neutrophil injections 24 h following the visualization of the plug. On gestational day 5.5, a decrease in implantation sites was observed.

Furthermore, on gestational day 18.5, there was a reduction in pregnancy rate and index, along with the presence of abnormal placental structure. Additionally, a reduction in the number of giant trophoblast cells and spongiotrophoblast cells was observed. These data emphasized the relationship between neutrophils and successful pregnancy [92].

In this comprehensive study, we developed a ceRNA network encompassing 29 DElncRNAs, 13 DEmiRNAs, and 18 DEmRNAs (Fig 6A). The ceRNA network contained hub and immune mRNAs (*CXCL11*, *EGR1*, *EGLN3* and *PRG2*) were visualized in Sankey plot (Fig 6B). Previous studies have revealed that the lncRNA *ERICH6-AS1* was a novel immune-related lncRNA in predicting cervical squamous cell carcinoma and endometrial carcinoma [93, 94].

Here, we screened possible drug candidates for PCOS through the CMap web tool. Enalapril is an angiotensin-converting enzyme (ACE) inhibitor, that hinders blood pressure. Prior research has indicated that PCOS women who maintain a body weight within the normal range tend to display higher blood pressure readings when contrasted to PCOS women. Moreover, enalapril could decrease the blood pressure in PCOS rats [95, 96]. Our results also suggested that the potential therapeutic candidacy of RS-100329, an adrenergic receptor antagonist, for PCOS treatment is being investigated. Propranolol (PROP), an adrenergic receptor antagonist, could prevent isoproterenol-induced polycystic ovary, enhancing the ovulation rate and reducing tyrosine hydroxylase and dopamine β-hydroxylase concentrations in the ovarian tissue [97, 98]. Numerous investigations have demonstrated that the NF-κB pathway contributes to PCOS development. Women with PCOS had elevated NF-κB levels in serum [99, 100], granulosa cells [101], endometrial tissue, endometrial cells, and mononuclear macrophages [102–104]. We identified Ro-106-9920, an NF-κB inhibitor, may lower PCOS NF-κB level. One study disclosed that phosphodiesterase 4 inhibitor plus metformin was superior to metformin alone in decreasing the testosterone levels in PCOS model rats [105]. Cilostamide is one of the phosphodiesterase inhibitors. The disrupted morphology of ovarian tissue and increased number of degenerated follicles were restored to normal levels after HDAC inhibitor treatment [106]. An HDAC inhibitor, tubacin, is also involved in the candidate drugs for PCOS therapy.

## Conclusions

Taken together, we assessed the immune status of women with PCOS at the end of pregnancy and explored possible molecular mechanisms. Herein, we comprehensively analyzed of the expression profiles of mRNAs, miRNAs, and lncRNAs derived from fetal side placental tissues. Our investigation focused on PCOS women as well as a control group. Additionally, we present the constructed ceRNA networks based on our findings. Furthermore, hub-DEmRNAs were retrieved, and KEGG, GO, and GSEA analysis revealed that immune systems, especially chemokines, were highly linked to PCOS pathogenesis. These outcomes would offer novel insight into the molecular mechanisms in the pathophysiological development of PCOS. Moreover, several small-molecule drugs that could be potential therapies for PCOS were screened.

## Supporting information

S1 Fig Functional enrichment analysis of DEmRNAs in fetal side placental tissue of PCOS and control group. (A) Bubble plot of enriched top 20 cell components (CC) terms ranking by rich factor. Enriched terms of (B) B cell receptor signaling pathway and (C) natural killer cell mediated cytotoxicity in Gene Set Enrichment Analysis (GSEA) analysis.
(TIF)

S2 Fig The validation of DERNAs of the ceRNA network related to the immune system in PCOS and control group using qRT-PCR. The quantification of mRNA, lncRNA, and miRNA expression levels was conducted utilizing the 2- ^ΔΔCT^ approach. GAPDH was used as reference gene for mRNA and lncRNA, and 5S was used as reference gene for miRNA. Data were reported as means ± SEM; n = 30 in each group. Two-tailed student’s t-tests were used, and significant differences were considered when the P-value < 0.05. *P < 0.05, **P < 0.01, ***P < 0.001, ****P < 0.0001.
(TIF)

S1 Table GSEA analysis of DEmRNAs in fetal side placental tissue of PCOS and control group. (XLSX)

S2 Table Cis regulated lncRNA and mRNA in fetal side placental tissue of PCOS and control group. (XLSX)

S3 Table Primer sequences for quantitative real-time polymerase chain reaction analysis. (DOCX)

S4 Table Prediction of potential drugs for the treatment of PCOS. (XLSX)
