# Supplementary figures and images for: Immune dysfunction mediated by the competitive endogenous RNA network in fetal side placental tissue of polycystic ovary syndrome
Source: PLoS One. 2024 Mar 21;19(3):e0300461. doi: 10.1371/journal.pone.0300461 (PMC10956758; doi:10.1371/journal.pone.0300461)

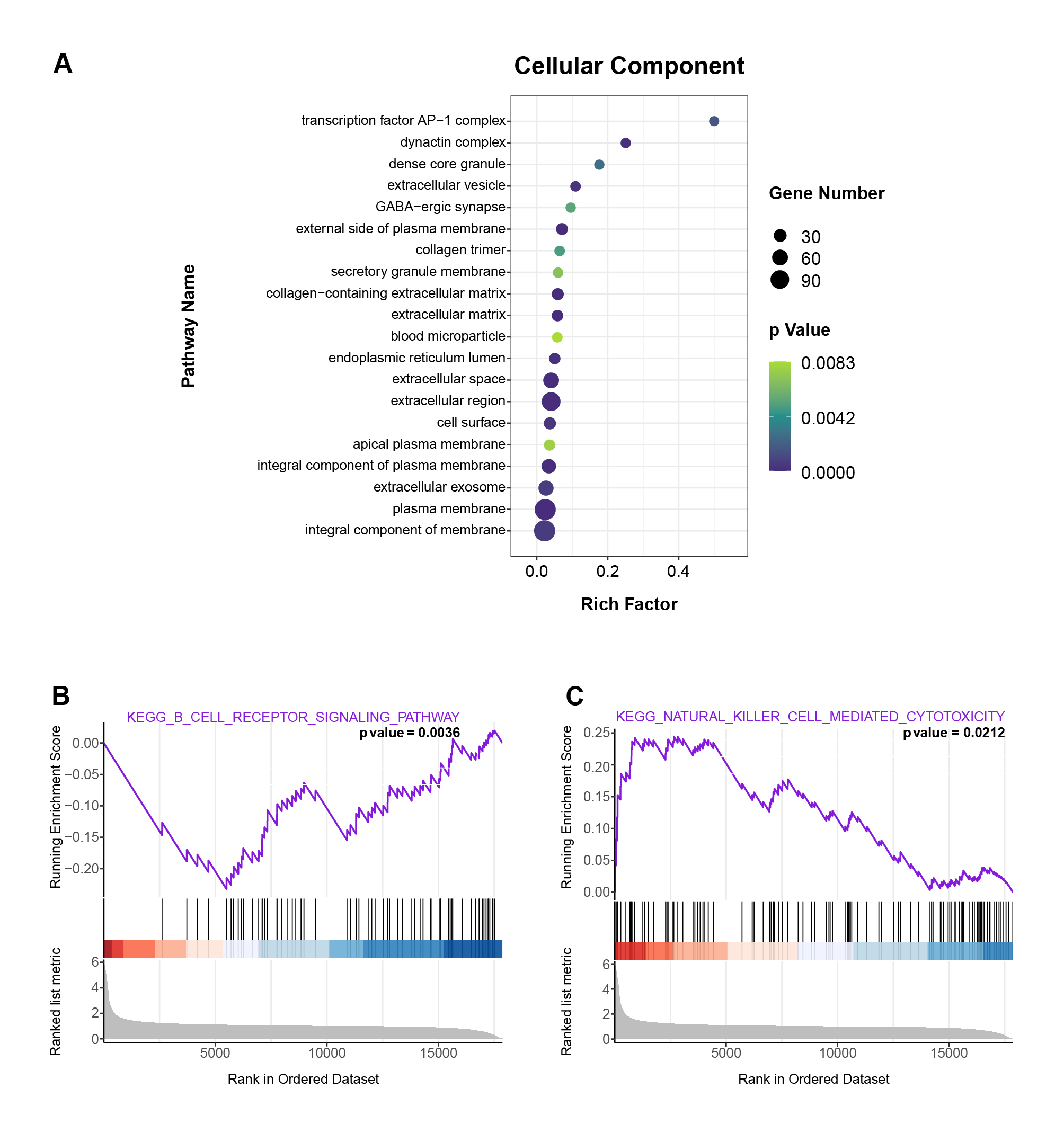

Supplement: S1 Fig — (A) Bubble plot of enriched top 20 cell components (CC) terms ranking by rich factor. Enriched terms of (B) B cell receptor signaling pathway and (C) natural killer cell mediated cytotoxicity in Gene Set Enrichment Analysis (GSEA) analysis. (TIF) [file pone.0300461.s001.tif]

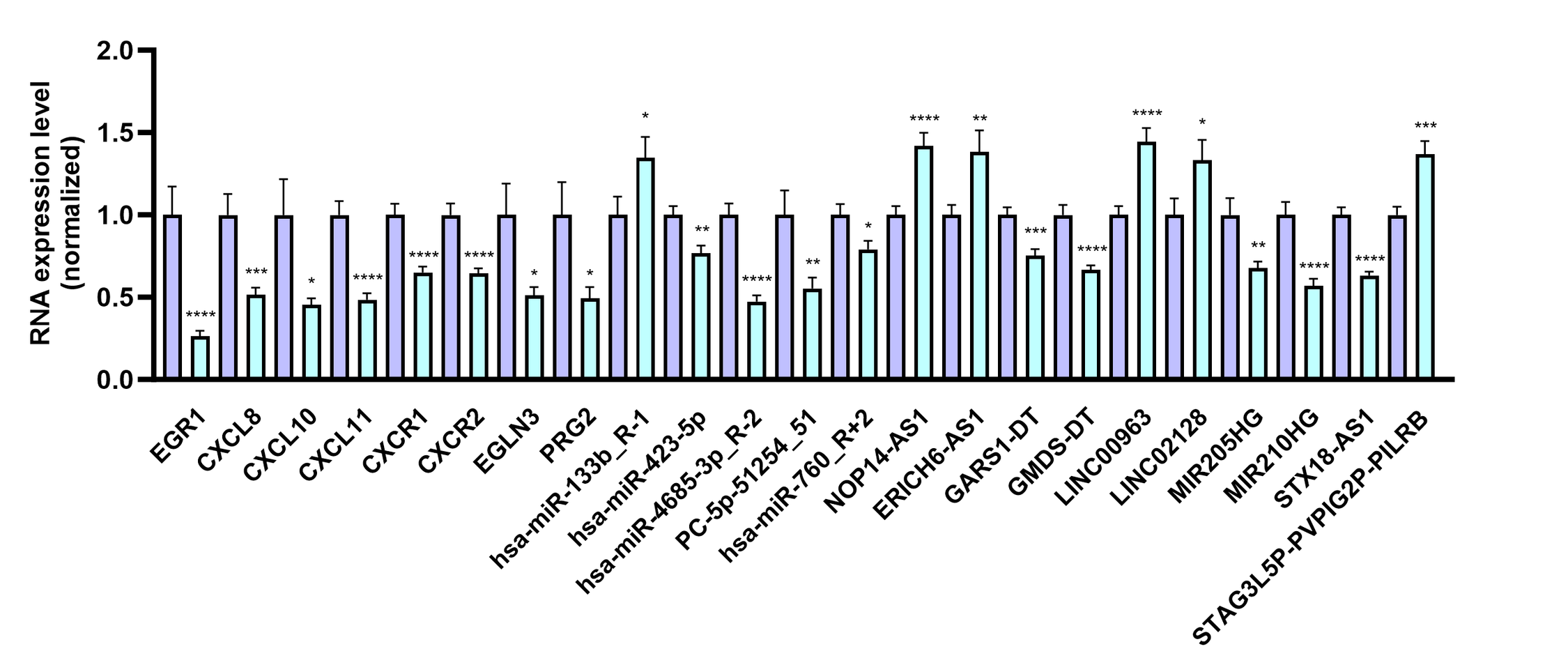

Supplement: S2 Fig — The quantification of mRNA, lncRNA, and miRNA expression levels was conducted utilizing the 2- ΔΔCT approach. GAPDH was used as reference gene for mRNA and lncRNA, and 5S was used as reference gene for miRNA. Data were reported as means ± SEM; n = 30 in each group. Two-tailed student’s t-tests were used, and significant differences were considered when the P-value < 0.05. *P < 0.05, **P < 0.01, ***P < 0.001, ****P < 0.0001. (TIF) [file pone.0300461.s002.tif]
