# Supplementary material for: Immune dysfunction mediated by the competitive endogenous RNA network in fetal side placental tissue of polycystic ovary syndrome
Source: PLoS One. 2024 Mar 21;19(3):e0300461. doi: 10.1371/journal.pone.0300461 (PMC10956758; doi:10.1371/journal.pone.0300461)
Supplement: S3 Table — (DOCX) [file pone.0300461.s005.docx]

Table S3. Primers used for quantitative real-time PCR analysis.

|  |  |  |
| --- | --- | --- |
| **RNAs** | **Forward primer (5′ to 3′)** | **Reverse primer (5′ to 3′)** |
| EGR1 | GGTCAGTGGCCTAGTGAGC | GTGCCGCTGAGTAAATGGGA |
| CXCL8 | ACTGAGAGTGATTGAGAGTGGAC | AACCCTCTGCACCCAGTTTTC |
| CXCL10 | GTGGCATTCAAGGAGTACCTC | TGATGGCCTTCGATTCTGGATT |
| CXCL11 | GACGCTGTCTTTGCATAGGC | GGATTTAGGCATCGTTGTCCTTT |
| CXCR1 | GGAGTTCTTGGCACGTCATC | ACCTTCCACACACAACCTCA |
| CXCR2 | GGCTTGATCAGCAAGGACTC | TGCACTTAGGCAGGAGGTCT |
| EGLN3 | TCCTGCGGATATTTCCAGAGG | GGTTCCTACGATCTGACCAGAA |
| PRG2 | AAACTCCCCTTACTTCTGGCT | GCAGCGTCTTAGCACCCAA |
| NOP14-AS1 | CTCGTTTCCTCTTTGCTTGG | TGCACGGATCCACAAATCTA |
| ERICH6-AS1 | CATGAGGAAGGAGGAGCAAG | ACTTGGACCCAAATGACTCG |
| GARS1-DT | CCAGGTCACCCCACTTCTAA | CAGCTGGACACAGAAAACGA |
| GMDS-DT | CCACTGCAAGACCAGAGTGA | ATCTGGAGAAGCCCCTCAAT |
| LINC00963 | GGTAAATCGAGGCCCAGAGAT | ACGTGGATGACAGCGTGTGA |
| LINC02128 | TGCCCCTAAAATCTGGTCTG | GGGTTCTACATGGCCTCTCA |
| MIR205HG | GAAGTTCAGGAGGCATGGAG | CTGAAGAAGCACGCACACTC |
| MIR210HG | CTATGCATTCCAGGCTCCAT | TCGGCTTGGTTATTTCTTGC |
| STX18-AS1 | TTGCCAACACAGACTCGAAG | AACAAAGTGGCGGAATTGAG |
| STAG3L5P-PVPIG2P-PILRB | TCCCAAGTGGTACATCGTCA | GAGTAGACAGCGGATGCACA |
| ACTB | CGACAGGATGCAGAAGGAG | ACATCTGCTGGAAGGTGGA |
| GAPDH | GGAGCGAGATCCCTCCAAAAT | GGCTGTTGTCATACTTCTCATGG |
|  |  |  |
